# Editorial

**DOI:** 10.1120/jacmp.v4i4.2495

**Published:** 2003-09-01

The great historian David McCullough (not related to me as far as I know) has said the study of history allows you to learn by your mistakes and plan for the future. At this juncture of time, that is good advice as far as JACMP is concerned.

Started in 1999 by the American College of Medical Physics (the first volumes were put online in 2000), this issue (Volume 4, issue 4, also called Fall 2003) is issue number 16 for those who are counting. The first three volumes (12 issues) were under the able stewardship of Peter Almond and Georgeanne Moore located in Houston, Texas, while this volume has been handled out of Rochester, Minnesota by Sarah Evers and myself. The JACMP survived this editorial transition but like the TV show, “Survivor,” 2003 was the year of big surprises.

The original goal of the ACMP–to have a place for papers more clinical than usually would get into other journals as well as being edited by physicists–has been achieved. What has not been achieved is the accomplishment of the original self‐supporting business models clearly set forth in 1998. The realities of supporting the JACMP by an organization with little growth and increasing costs of its other obligations has hit home, and the ACMP Board decided to terminate its publishing contract with AIP. Prior to this decision an ad hoc committee made up of very knowledgeable and luminous persons got together to see what made sense. Representing the AAPM were Chris Marshall, Colin Orton, and John Boone, while the ACMP was represented by Jim Smathers, Ned Sternick, and myself. Melissa Martin (AAPM treasurer) also attended the meeting in College Park.

The basic result of these meetings was to recommend that the AAPM seriously consider the concept of being a quarterly, online supplement to the journal Medical Physics, containing “clinical” papers. Implicit in this approach were a number of desirable factors:

1. Transition the papers already in the JACMP editorial process to the first issue;
2. For the first two years try to have a separate Editorial Board;
3. Develop a clear editorial policy that would guide authors as to where submissions made sense as well as insure the reader got the type of material expected;
4. Preserve the first four years of JACMP.

I can report to you that this plan has yet to receive any action, and the pessimists in the audience could easily walk away with the impression that this JACMP issue (Volume 4, No. 4) is the last issue. Such a view would be a shame for a number of reasons, including the obvious one of loss of visible authorship by those who have published already. More important, I believe, is the original view that there needed to be a physicist peer‐reviewed journal that has a clear and consistent policy of publishing papers that are immediately applicable in patient care situations. I would also argue this type of format is ideal for publishing refresher course notes, task group reports, how‐to articles, or collections of data (e.g., CTDI data from a variety of CT scanners). It is unrealistic to do this in a paper format, but certainly the electronic format offers an economy that guarantees a good return for the dollars invested.

Rumors about JACMP swirl like the snow in Minnesota. When the Spring of 2004 comes we will have to see what emerges from underneath the blanket of snow. Will it be winter killed grass or a beautiful crocus pushing up through the snow? I vote for the latter, but a God I am not.

As a final action, I must thank in a very sincere manner all those that served with me on the Editorial Board. The job is thankless, and all members risk being badgered by yours truly. But they understood the goals and high standards we have set. It has been an honor to lead you.

Edwin C. McCullough

Editor‐in‐Chief

